# Designing a Mobile App for Post Traumatic Stress Disorder: Insights From Lived Experience and Clinical Practice

**DOI:** 10.1049/htl2.70098

**Published:** 2026-10-01

**Authors:** Richard Laugharne, Michael Loizou, Marius Varga, Sivia Lin, Sarah Lennard, Bojana Daw Srdanovic, Roger Farrell, Rohit Shankar

**Affiliations:** ^1^ CIDER University of Plymouth Truro Truro UK; ^2^ Cornwall Partnership NHS Foundation Trust Truro UK; ^3^ Tycho Medlink LTD London London UK

**Keywords:** co‐production, digital health intervention, lived experience, mobile health application, post‐traumatic stress disorder

## Abstract

Post‐traumatic stress disorder (PTSD) is a disabling anxiety disorder precipitated by severe trauma. Many people with this condition have little access to clinical interventions, especially in low‐middle‐income countries. For the development of any digital health intervention, it is vital to know the opinions of PLE and clinicians to guide the developers. We conducted two focus groups, one with clinicians (*n* = 5) and one with people with lived experience (PLE) (*n* = 11), to gain knowledge of their views on the development of a PTSD App. Transcripts of both focus groups were coded and thematically analysed. The key themes that emerged from the PLE group were the importance of ease of use with clear, brief, positive but not patronising communication; control over the App styling; and the desire for a variety of interventions and outcome measures. For the clinical group, main themes included bite‐sized positive communication, App providing a choice of interventions and outcome measures while monitoring progress. Both groups talked positively about how gamification might be helpful. Our small study highlights that co‐producing health Apps, such as the proposed PTSD one with lived experience and clinicians, ensures accessible, non‐patronising design, meaningful choice, and scalable digital support to extend care where services remain limited.

## Graphical abstract

This qualitative study explored clinician and lived‐experience perspectives to inform the co‐development of a digital intervention for post‐traumatic stress disorder (PTSD), particularly relevant where access to care is limited. Focus groups with clinicians and people with lived experience highlighted the need for an easy‐to‐use, non‐patronising app offering user control, choice of interventions and outcomes, progress monitoring, and potential gamification. Co‐production with both groups may support the development of accessible, scalable digital tools to extend PTSD care in resource‐constrained settings.

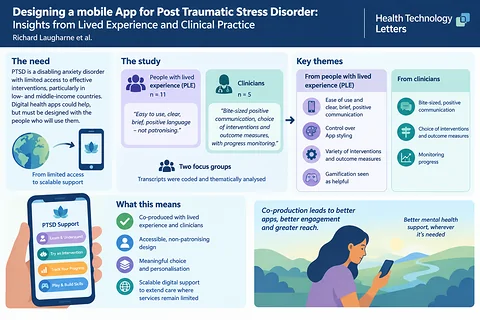

## 1 Background

Post‐traumatic stress disorder (PTSD) is an anxiety disorder precipitated by severe trauma and characterised by nightmares and flashbacks of the traumatic incident, hyper‐vigilance in situations reminding the person of the events and autonomic arousal in situations connected with these events [1]. A minority of people who experience trauma go on to develop PTSD [2]. Between 2 and 5% of the population are thought to experience PTSD at any one time [2].

Antidepressant medication is the most used treatment for PTSD and is considered effective even though they are recognised as being second line of management [3, 4]. There are evidence‐based treatments for PTSD, including certain talking therapies [4, 5, 6]. Trauma‐focused psychological interventions have been considered superior to pharmacological interventions for PTSD [4, 7]. Small mean effect sizes of less than 0.4 are found for the most successful drugs and large mean effect sizes of over 1.2 for trauma‐focused cognitive behavioural therapy (T‐fCBT) and eye movement desensitisation and reprocessing (EMDR) [7]. Medication also has shown potential for significant side effects and the risk of relapse of symptoms after stopping and is generally regarded for managing symptoms rather than directly processing the trauma itself [4, 6]. The reason why anti‐depressants are used more widely is possibly due to ease of availability, access and cost compared to therapy [8]. The World Health Organisation (WHO) estimates that 80% of people with PTSD globally have little or no access to treatment, particularly therapy, especially in low‐middle income countries [2].

There is a growing evidence base for mobile interventions in PTSD, with systematic reviews published [9, 10]. However, studies are often limited by small sample sizes, brief intervention, and follow‐up periods, and self‐reported measures of PTSD [9], and the later systematic review concluded there was a low strength of evidence for their effectiveness [10]. No study has meaningfully looked at views of people with lived experience and clinicians to consider the utility of a PTSD app.

Digital interventions offer a potentially scalable means to broaden access to PTSD support by delivering self‐guided psychoeducation, symptom management tools, and therapeutic exercises directly to users without reliance on limited local clinical infrastructure, helping overcome barriers to in‐person care that are common in low‐resource settings [11]. Smartphone Apps like PTSD Coach have been shown to reduce PTSD symptoms and improve self‐management outcomes compared with waitlist control groups, highlighting their potential as widely accessible public health interventions [12].

In resource‐constrained environments, adapting mobile PTSD tools with contextual support can increase acceptability, feasibility, and engagement, helping to widen access to care where formal mental health services are scarce [13]. These interventions can also facilitate task‐sharing by supporting non‐specialist providers and paraprofessionals to deliver or augment evidence‐based PTSD treatments, thereby addressing workforce shortages prevalent in many low‐ and middle‐income countries [13]. Although evidence is still emerging and few Apps have been rigorously evaluated, mobile health technologies represent a promising avenue to bridge treatment gaps and extend PTSD therapeutic resources to underserved populations globally [11].

Digital interventions often fail due to low user engagement, a problem frequently linked to the lack of involvement of people with lived experience (PLE) and clinicians during the design and development phases. This results in products that do not align with real‐world needs and clinical workflows [14].

Thus, it is important from the earliest stages of development of any digital device for PTSD to include and involve PLE to guide the researchers in designing such an App [15]. However, there is limited evidence of current technology being co‐produced by PLE and clinicians. In our study we sought to understand the views of PLE of PTSD and their clinicians of their views of developing an App for PTSD management.

## 2 Methods

### 2.1 Study Population

Specialist clinicians known to be experts in PTSD were recruited from known contacts of the authors. PLE were recruited through a national charity, PTSD UK. The charity informed their members of the research project with an email to contact the research team if they wished to take part. People who emailed the team were contacted by a study coordinator. The study was described, and the caller then decided if they wanted to take part in the focus group or not.

### 2.2 Study Design

The focus groups were conducted online, virtually, by Microsoft Teams and led by the first author. Each focus group lasted 1.5 h. The research team prepared a semi‐structured questionnaire for both groups to generate conversation. The questions were developed collaboratively by the authors and were intended to focus on the development and utility of the potential App. These questions are listed in supplementary information 1. In addition, an early prototype of the App was developed and demonstrated to the participants to stimulate discussion (https://www.youtube.com/watch?v)

### 2.3 Ethics and Governance

Ethics approval was obtained on 13/09/2024 from the University of Plymouth (Project ID 5024: PTSD). All participants were advised at the start of the study that participation was voluntary and their replies, if they chose to participate, would be anonymised and analysed. Verbal informed consent was taken from all participants when attending the groups. No participant identification data was collected. Participant information about the study was presented verbally at initial contact and repeated at the start of the focus group sessions. The intention of the study was to enable those taking part to participate in co‐production as experts by experience.

### 2.4 Analysis

The two focus group sessions were recorded and transcribed automatically. The transcripts were coded in NVivo by two qualitative researchers who had not taken part in the focus groups, working independently to provide researcher triangulation. Coding was done inductively within the broad structure established by the semi structured questionnaire. The researchers then compared and combined the separate code structures to make one unified code structure, resolving disagreements through discussion. This code synthesis generated 12 unique codes for the Clinician focus group and 29 for the PLE focus group. Thematic analysis was then undertaken by the independent researchers in consultation with the lead author to structure and interpret the data.

## 3 Results

Clinicians included four full‐time working‐age psychiatrists (three males, 1 female) and one female nurse specialist, all of whom had extensive experience in treating people with PTSD. Eleven people with lived experience (PLE) of PTSD agreed to take part in the focus group, having responded via the charity PTSD UK. Nine were female and two were males. The age range was from 28 to 64 years. All reported experience of PTSD. We present results with the themes emerging from PLE and clinicians, respectively, and select quotes from the participants as examples.

### 3.1 People With Lived Experience

PLE approached the discussion broadly through two lenses which were integrated throughout: their experiences of PSTD and therapy; the actual and potential impact of digital apps on their experiences. Table 1 provides the themes and sub‐themes which are further discussed below.

**TABLE 1:** Themes from People with lived experience.

Themes	Subthemes/codes synthesised from independent analysis
**Experiences** & relevant symptoms of PTSD	Catastrophising Co‐occurring conditions Emotional numbness Intrusive thoughts Triggering
EMDR **Experiences**	EMDR Experiences
**Experience** & app relevance of direct therapy (general)	Therapies for app to consider Therapy technique—Breathing Therapy technique—Meditation App as an alternative to therapy Family
Concerns about a PTSD **app**	Existing Apps drawbacks—Exacerbating symptoms Apps Comparison Cost Features—Avoid Data Protection Logging (Repeating) Symptoms
Potential for a PTSD **app**	Features—Preferred Education Peer support Existing Apps Positive Mapping Mood & Feelings
EMDR **App** Challenges	EMDR App Challenges
**App** Features—Things to consider	App layout and accessibility International considerations Moderation and Safety Tracking Progress—Feedback
Games and gamification in **app**	Games

#### 3.1.1 Experiences & Relevant Symptoms of PTSD

Several participants referred to the impact of PTSD symptoms on their lives, including emotional dysregulation and a corresponding emotional numbness or inability to describe their emotional state, sensitivity to triggers, challenges with Catastrophising and intrusive thoughts. Some also noted how co‐occurring conditions, such as autism and fibromyalgia, interrelate.

#### 3.1.2 EMDR Experiences

EMDR is an evidence‐based therapy for PTSD recommended by the National Institute of Clinical and Social Excellence [4]. Some participants had found face‐to‐face EMDR had made their symptoms worse; some found it intense and even brutal, whilst others had been helped by butterfly tapping but not eye movements.

#### 3.1.3 Experience & App Relevance of Direct Therapy (General)

Other therapies were spoken about positively and suggested as part of the App. These included breathing exercises, self‐soothing exercises, compassion‐focused exercises and safety planning/grounding. Participants also talked positively about inter‐family relations therapy and neurofeedback. It was suggested that games could be used to positively enable neurofeedback. Simple therapeutic tools such as words and pictures were also positively suggested.

#### 3.1.4 Concerns About a PTSD App

The cost of any App was important to the participants so that it was not too expensive for them to use. The participants did not want the App to be too ‘wordy’. Being bombarded with information can be overwhelming, and it is important that the communication style is not patronisingly positive. There was also a desire that the App could include content that would stop automatically, as participants can use material to fall asleep too. Any chat between users would need to be moderated to ensure there is no inappropriate behaviour.

'I have used other mental health apps and then got put off quite quickly because they are so overwhelming with the amount of information that's on there. You know, like even simple meditation….you just, you just don't know where to start. It's just, it's too much and I've now got **** app. It literally has three meditations on that's it. Because it's enough. You just don't want to be bombarded, I would say’.(PLE 2)

'*I've never ever really got on with them, often because I really feel they talk down to you. I hate apps that kind of go well done, you're doing really great….and it's just like, no, no, actually I'm not doing really great right now or whatever. But…I know what they're trying to do. It's trying to be sort of really positive, but sometimes….it comes across as you're 4 years old and….if it's if it's designed for adults, I think it should be on an adult level’. (PLE 3)*

Some participants find having to describe their own symptoms traumatising, and it can cause anxiety if possible symptoms are described.

#### 3.1.5 Potential for a PTSD App

The group believed that the App could create a safe community that could provide peer support for people suffering from PTSD. They would very much appreciate the App being free to use.

*'Because there's no PTSD support groups, but any sort of way that we can get peer support. I think somebody's already mentioned, you know, putting in tips and things like that, but if there is any way within the app where you can have, I know that it that might be quite difficult, but you can chat with others who are going through similar things that makes such a massive difference to me if ever I have any contact with anyone who has PTSD…. that's definitely something I desperately would love, would be some sort of peer support’. (PLE 4)*

The participants desired ways of self‐monitoring, including patterns of mood and a visual scale of emotional energy. They expressed a wish for educational materials on brain physiology, mindfulness, pain and physical symptoms and emotional numbness. They wanted enough information so that they could make informed choices.

*'One of the most important things for me was psychoeducation. And have somebody explain the physiology of the brain and how it gets changed in PTSD? And without that, I couldn't until somebody did that and having that really simple because obviously I'm a scientist, so I can understand it, but it seems to me it's quite complicated. So having a simple version of that, that's not too patronising’. (PLE5)*

#### 3.1.6 EMDR App Challenges

PLE were concerned that if this was delivered by the App there may be issues of safety. There was a clear nervousness in some, but not everyone agreed, and some were more positive about EMDR, so there were differences in the group.

#### 3.1.7 App Features—Things to Consider

The group valued the prototype being simple to use and easy to navigate. They cared about the design, including the use of colours to help. They expressed a desire for some ability to self‐design: by choosing the colours for their App and adding their own material such as pictures, videos and quotes, as well as controlling the font and having light and dark options.

*'For me a good app almost needs to be able the person's going to use it almost be able to set up that screen format to suit them in some way. I don't know how you do that, but that would be my ideal on an app’*.

*(PLE 1)*

The participants wanted to control when they saw their feedback and did not want notifications. It was felt that seeing feedback when things are not going well can lead to self‐criticism and despair. Ideally users should choose what feedback tools are used from a range, and these should be both positive (strengths) and negative (symptom scales) in nature. Participants preferred visual presentation of scales over numeric.

#### 3.1.8 Games and Gamification in App

Some participants suggested using games both to make the app fun and to deliver significant benefits, referring to the established grounding potential of Tetris, for example.

### 3.2 Clinicians

Table 2 provides the themes and sub‐themes which are further discussed below.

**TABLE 2:** Themes from Clinicans.

Themes	Subthemes/codes synthesised from independent analysis
Existing context—Relevant experience of PTSD therapy	Other DIs and physical resources De‐pathologising PTSD Interventions—Negative experiences International context
Value of an App approach	Purposes of App App Experience App features (other than or supporting EMDR) Humour Monitoring
EMDR—Key aspects and how to include in an app	EMDR Clinicians’ perspective EMDR on App
Gamification	Gamification

#### 3.2.1 Existing Context—Relevant Experience of PTSD Therapy

Some existing DIs, such as CBT based modules used in some NHS settings, lack personalisation potential. A positive message needs to be given through the App, avoiding retraumatising the patient and preferably not being too structured, using positive language such as ‘What happened to you?’ rather than “what is wrong with you?,” and ‘you will survive, you are not sick’.

‘*But I also think talking to it again, as in not what's wrong with you, but what happened to you, but in a very empowering….this isn't about amplifying the sick role, but actually saying you're an extremely capable, competent human being who's had something horrific happen to you and you survived it. And if you can survive that, you'll be able to survive any processing that you do.’ (Clinician 2)*

#### 3.2.2 Value of an App Approach

When using Apps, the positive attributes of useful apps experienced by clinicians were regular invitations to use the App to remind the user and that advice given is in small, bite‐sized chunks rather than being too wordy. Clinicians liked engaging cartoons with humour, and clinicians like joining with others and creating communities online.

*'*‘*They have lots of little cartoon snippets, just giving little lovely gems of bits of advice and I think it's really quite useful ….when things are just in little bite size pieces and you can just keep ….approaching the app and getting more and more little gems like that, gems of wisdom’.(Clinician 1)*

Negative aspects identified focused on boring content that was too much like homework at school. The clinicians also felt the cost of the App was important for patient engagement.

*'But I'm just thinking it's got to be really easy to access, user friendly and all the things we've heard tonight about graphics ….to get away from that sort of overwhelming sort of what we said earlier on about….just like being in a classroom, getting away from that’. (Clinician 3)*

The monitoring of progress needs to focus on positive outcomes in the opinion of the clinicians. Symptom monitoring can be pathologising and depressing, whereas the monitoring functioning and quality of life more positive. Subjective progress scales and wellness scales could be used. Setting goals and monitoring progress to that goal can be helpful.

#### 3.2.3 EMDR—Key Aspects and How to Include in an App

The clinicians were positive about using EMDR and also identified the benefits of the butterfly hug technique, the flash technique, stacking the deck and the double sigh, all techniques used in EMDR. They felt that some of these could be gamified for an App, such as using flower petals for stacking the deck. For the safe place technique, it was important for the user to be able to upload their own photos or drawings. The importance of empowerment and choice was emphasised.

#### 3.2.4 Gamification

Possible new content developments include gamification, providing users with greater choice where they could choose from a range of modules so that it is not ‘like school’.

‘*But I think ….that the ethos of this app is about choice. Because if there's any sense from a patient that they've got to go through a process and it's rigid, that can often be quite re‐traumatising or… quite difficult for people and so inviting there to be lots of choice throughout the whole process…to personalise it too. Invite people….to choose the right time. I just think choice is… such a vital part of whatever app we pick*‘*. (Clinician 1)*

Other gamification suggestions included using character avatars to set and monitor goals and provide prompts, and using cartoon elements to enhance exercises like the EMDR container exercise.

## 4 Discussion

This study explored the perspectives of PLE of PTSD and specialist clinicians regarding the development of a mobile application to support PTSD self‐management. The findings highlight the complexity of digital interventions in this population, underscoring the importance of co‐production, personalisation, and safety in the design of PTSD‐focused digital tools.

### 4.1 Alignment With Evidence‐Based PTSD Care

Consistent with existing literature, participants emphasised the value of psychoeducation, grounding techniques, breathing exercises, and self‐soothing strategies—interventions that are well aligned with first‐line trauma‐focused psychological approaches for PTSD [4, 5, 6, 7]. PLE expressed a strong desire for accessible explanations of the neurobiology of PTSD, supporting evidence that psychoeducation can enhance self‐efficacy, reduce stigma and improve engagement with treatment [16, 17]. The preference for concise, non‐patronising content delivered in manageable ‘bite‐sized’ units reflects best practice in digital mental health design and mirrors findings from other App evaluations [14].

Notably, both PLE and clinicians stressed that digital tools should complement rather than replace trauma‐focused therapies. This is particularly relevant given evidence that pharmacological treatments have smaller effect sizes compared with trauma‐focused cognitive behavioural therapy (TF‐CBT) and EMDR and are often used due to access constraints rather than clinical superiority [4, 7]. The findings support the positioning of mobile apps initially as adjunctive or preparatory tools that can enhance readiness, self‐regulation, and coping outside formal therapy sessions.

### 4.2 Engagement, Usability and Personalisation

Low engagement is a recognised limitation of many digital mental health interventions [14, 18]. Participants in this study clearly articulated design features that they believed would sustain engagement, including simplicity, aesthetic control, optional feedback, and the avoidance of overwhelming or overly didactic content. The desire for customisable interfaces, including colour schemes, fonts, light/dark modes, and personally meaningful media, aligns with emerging evidence that personalisation increases perceived relevance and sustained use of mental health Apps [19].

Importantly, PLE strongly rejected patronising language and unsolicited notifications, echoing concerns raised in prior qualitative studies that poorly designed digital feedback can exacerbate shame, self‐criticism and disengagement in trauma survivors [20]. Clinicians similarly emphasised choice, flexibility, and empowerment as core principles, reinforcing trauma‐informed care frameworks that prioritise autonomy, collaboration, and emotional safety [21].

### 4.3 Peer Support and Community

PLE identified peer support as a potentially powerful component of the App, particularly in the context of limited access to formal PTSD services. This is consistent with evidence that peer support can reduce isolation, normalise experiences and improve coping in mentally unwell populations [22]. However, participants also highlighted the need for careful moderation to prevent harm, misinformation or re‐traumatisation. This mirrors wider concerns in digital mental health regarding governance, safeguarding, and ethical responsibility in online communities particularly in low‐middle‐income countries [21, 23].

### 4.4 EMDR, Safety and Digital Delivery

A key area of divergence between PLE and clinicians is related to the inclusion of EMDR‐based techniques within the App. While clinicians viewed certain components (e.g., butterfly hug, safe place and flash technique) as potentially amenable to digital or gamified delivery, some PLE reported that EMDR had previously been distressing or destabilising. This highlights a critical tension between innovation and safety. Although early studies suggest that digitally supported EMDR elements may be feasible, there is currently limited evidence regarding their safety and effectiveness when delivered without therapist support [24, 25].

These findings reinforce the need for robust safety features, clear boundaries, optional engagement, and explicit guidance that such tools are not substitutes for therapist‐led trauma processing, at least till a better evidence base is available. The expressed concern around ‘target selection’ for trauma memories further underlines the complexity of translating therapist‐mediated interventions into self‐guided digital formats.

### 4.5 Monitoring, Outcomes and Reframing Recovery

Both groups expressed reservations about symptom‐focused monitoring, with concerns that repeated symptom tracking could feel pathologising or demoralising. Instead, clinicians advocated for monitoring quality of life, functioning, and goal attainment, an approach supported by recovery‐orientated and trauma‐informed models of care [26, 27]. PLE similarly preferred visual, optional, and user‐controlled feedback mechanisms. These preferences align with growing calls to move beyond symptom reduction as the sole marker of recovery in PTSD and to incorporate wellbeing, resilience, and meaning‐based outcomes [28].

### 4.6 Co‐Production as a Methodological Strength

A central contribution of this study is the demonstration of the value of involving both PLE and clinicians at an early stage of digital intervention development. The findings provide concrete examples of how co‐production can identify potential harms, enhance acceptability, and guide design choices that align with real‐world needs. This addresses a recognised gap in the digital mental health literature, where many interventions fail to demonstrate meaningful stakeholder involvement despite strong evidence that co‐produced interventions achieve higher engagement and relevance [15, 29].

### 4.7 Limitations

This study has several limitations. The sample size was small, and participants were self‐selected, potentially introducing selection bias towards individuals with strong views or prior engagement with digital tools. Participants were from the UK and Australia and so limited to these cultural milieus. The focus groups were conducted online, which may have excluded individuals with limited digital literacy or access. The clinician group is mostly psychiatrists (4/5), with only one nurse specialist. Other key perspectives from psychologists or therapists are missing. The lived experience group is small, largely female, and recruited via a single UK charity. This raises questions about representativeness and limits the generalisability of the findings. Additionally, as this was an exploratory qualitative study, findings cannot be generalised without further evaluation in larger and more diverse populations and are indeed illustrative rather than generalisable.

It needs to be recognised that given that PTSD populations are particularly vulnerable, unsupervised trauma processing carries real risk. Safety and ethical considerations regarding trauma processing were not the focus of this study. Future projects, especially prior App development, need to consider which EMDR elements are not appropriate for self‐guided use as well as clarify that certain techniques would require external clinical support or be restricted to grounding‐only adaptations.

Several authors of this paper are involved in PTSD clinical care and digital intervention development, which could bring its bias to the research done. However, this was mitigated considerably by bringing three separate academics, two post‐doctoral researchers specialising in social sciences and one associate professor in digital health who had no previous involvement in the project. This reduced any direct influence of those authors previously involved in the project or having potential conflicts of interest in the App being positively supported.

## 5 Conclusion

### 5.1 Implications for Future Research and Practice

Future work should focus on iterative co‐design, usability testing, and formal evaluation of safety, engagement, and clinical outcomes. Particular attention should be paid to safeguarding, moderation of peer support features, and the ethical integration of trauma‐focused techniques. We hope that these findings can inform developers going forward in designing apps to help people with PTSD. There is a real requirement to reach out to the 80% of people in the world who cannot access treatment for PTSD. New technologies may offer some solutions to this treatment deficit.

## Author Contributions

**Richard Laugharne**: conceptualisation, formal analysis, funding acquisition, investigation, project administration, supervision, validation, visualisation and writing – original draft. **Michael Loizou**: conceptualisation, formal analysis, funding acquisition, investigation, project administration, supervision, validation, visualisation and writing – original draft. **Marius Varga**: data curation, formal analysis, methodology, visualization, writing – review and editing. **Sivia Lin**: data curation, formal analysis, methodology, visualization, writing – review and editing. **Sarah Lennard**: data curation, formal analysis, methodology, visualisation, writing – review and editing. **Bojana Daw Srdanovic**: investigation, validation, visualisation, writing – review and editing. **Roger Farrell**: investigation, validation, visualisation, writing – review and editing. **Rohit Shankar**: conceptualisation, data curation, formal analysis, funding acquisition, investigation, methodology, resources, validation, visualisation, writing – review and editing.

## Funding

The development of this paper was enabled by the EPSRC funded N‐CODE Network.

## Conflicts of Interest

RS has received institutional and research support from LivaNova, UCB, Eisai, Veriton Pharma, Neuraxpharm, Bial, Angelini, UnEEG and Jazz/GW pharma outside the submitted work. No other author has any declared conflict of interest related to this paper.

## Data Availability

All data used for the paper is within the manuscript.
